# Protective Effects of Carnosol on Renal Interstitial Fibrosis in a Murine Model of Unilateral Ureteral Obstruction

**DOI:** 10.3390/antiox11122341

**Published:** 2022-11-26

**Authors:** Jae-Hyung Park, Jaechan Leem, Sun-Jae Lee

**Affiliations:** 1 Department of Physiology, Keimyung University School of Medicine, Daegu 42601, Republic of Korea; 2 Department of Immunology, School of Medicine, Daegu Catholic University, Daegu 42472, Republic of Korea; 3 Department of Pathology, School of Medicine, Daegu Catholic University, Daegu 42472, Republic of Korea

**Keywords:** carnosol, renal fibrosis, oxidative stress, endoplasmic reticulum stress, apoptosis, necroptosis, inflammation

## Abstract

Renal fibrosis is a common feature of chronic kidney disease and is a promising therapeutic target. However, there is still limited treatment for renal fibrosis, so the development of new anti-fibrotic agents is urgently needed. Accumulating evidence suggest that oxidative stress and endoplasmic reticulum (ER) stress play a critical role in renal fibrosis. Carnosol (CS) is a bioactive diterpene compound present in rosemary plants and has potent antioxidant and anti-inflammatory properties. In this study, we investigated the potential effects of CS on renal injury and fibrosis in a murine model of unilateral ureteral obstruction (UUO). Male C57BL/6J mice underwent sham or UUO surgery and received intraperitoneal injections of CS (50 mg/kg) daily for 8 consecutive days. CS improved renal function and ameliorated renal tubular injury and interstitial fibrosis in UUO mice. It suppressed oxidative injury by inhibiting pro-oxidant enzymes and activating antioxidant enzymes. Activation of ER stress was also attenuated by CS. In addition, CS inhibited apoptotic and necroptotic cell death in kidneys of UUO mice. Furthermore, cytokine production and immune cell infiltration were alleviated by CS. Taken together, these findings indicate that CS can attenuate renal injury and fibrosis in the UUO model.

## 1. Introduction

Chronic kidney disease (CKD) is defined as the presence of decreased kidney function and/or kidney damage for at least 3 months, irrespective of the underlying cause [1]. The prevalence of CKD has steadily increased over the past 3 decades, becoming a global health problem [1]. CKD is also considered a risk factor for cardiovascular disease and is related to increased mortality [2]. The pathogenesis of CKD still remains incompletely understood despite intensive research because it is complex and involves multiple factors [3]. Renal fibrosis is a common hallmark of CKD and is characterized by an excessive deposition of extracellular matrix (ECM) [4]. Myofibroblasts express α-smooth muscle actin (α-SMA) and produce large amounts of ECM proteins during fibrosis [5]. Differentiation and activation of myofibroblasts are modulated by pro-fibrogenic cytokines including tumor growth factor-β (TGF-β) and connective tissue growth factor (CTGF) [5]. Current treatments for CKD include life modification, medication, dialysis and kidney transplantation [1]. However, specific treatments for renal fibrosis are limited, so the development of new anti-fibrotic agents is urgent.

Accumulating evidence suggests that oxidative stress plays a critical role in the development of renal fibrosis [6]. The kidney is the organ with the second highest requirement for oxygen consumption and mitochondrial contents [7]. Indeed, a large amount of ATP is required for the reabsorption of solutes by tubular epithelial cells. In pathological conditions, excessive reactive oxygen species (ROS) are produced, leading to progressive mitochondrial damage [7,8]. Damaged mitochondria exhibit a loss of efficiency of the electron transport chain, enhancing ROS generation and decreasing ATP production. The oxidative stress-induced mitochondrial dysfunction can perturb various cellular processes and contribute to the development of inflammation, tubular cell apoptosis, and fibrosis [7,8]. In addition, inflammation and tubular cell apoptosis further promote renal fibrosis. Therefore, anti-oxidative agents could be served as potential anti-fibrotic therapies for renal fibrosis.

Over the past few decades, natural products have played a critical role in drug discovery for a variety of human diseases, such as cancer, infectious diseases and cardiovascular diseases [9]. The use of natural products in drug development has several distinct advantages [10,11]. They exhibit chemical novelty and can provide lead drug candidates for complex targets compared to other sources. In addition, natural products have unparalleled chemical diversity compared to synthetic chemicals [10,11]. Even with a complex molecular structure, they can be absorbed and metabolized in the body. Accumulating evidence suggests that natural products with antioxidant properties, such as berberine and curcumin, have beneficial effects in preclinical models of CKD [6]. Carnosol (CS) is a natural diterpene compound found in rosemary plants and has several biological effects, including anti-tumor, antioxidant and anti-inflammatory properties [12,13]. This compound has been shown to exhibit protective effects on various inflammatory diseases, such as experimental autoimmune encephalomyelitis [14], inflammatory bowel disease [15], spinal cord injury [16], nonalcoholic fatty liver disease [17], allergic asthma [18] and atopic dermatitis [19]. Furthermore, CS ameliorated ischemia/reperfusion-induced acute kidney injury in rats [20]. However, the effect of CS on renal fibrosis has not yet been investigated

The unilateral ureteral obstruction (UUO) model is a well-established model of progressive renal interstitial fibrosis [21]. This model has been widely used to obtain new therapeutic agents for renal fibrosis [22]. In this study, we investigated the potential protective effects and underlying mechanisms of CS against renal fibrosis in the UUO mouse model.

## 2. Materials and Methods

### 2.1. Animal Experiments

Seven-week-old male C57BL/6J mice were purchased from HyoSung Science (Daegu, Republic of Korea). Before starting experiments, the mice were acclimated for 1 week under 20–24 °C on a 12/12 h light/dark cycle. Animal experiments were approved by the Institutional Animal Care and Use Committee of the Daegu Catholic University Medical Center (DCIAFCR-211220-30-Y). The mice were randomly grouped into four groups (*n* = 8 in each group): (1) sham-operated control (Sham) group; (2) Sham+CS group; (3) UUO group; (4) UUO+CS group. To establish UUO model, the left kidney was exposed through a flank incision under general anesthesia and the left ureter was ligated with 5-0 silk sutures. The sham-operated group underwent surgical procedure similar to UUO but not subjected to ureteral ligation. The Sham+CS and UUO+CS group were given intraperitoneal injections of CS (50 mg/kg, dissolved in DMSO) daily for 8 consecutive days, starting from 1 day prior to the sham or UUO operation. The Sham and the UUO group were injected intraperitoneally with an equal volume of DMSO. CS was purchased from Cayman Chemical (Ann Arbor, MI, USA). The dose of CS was chosen based on previous studies [14,15]. One week after the sham or UUO operation, all mice were anesthetized and sacrificed. Blood samples were collected by cardiac puncture and then the kidneys were rapidly isolated.

### 2.2. Determination of Creatinine, Blood Urea Nitrogen (BUN) and Cytokine Levels

Serum levels of creatinine and BUN were analyzed using an autoanalyzer (Hitachi, Osaka, Japan). Serum and renal levels of tumor necrosis factor-α (TNF-α), interleukin-6 (IL-6) and IL-1β were measured using ELISA kits (R&D Systems, Minneapolis, MN, USA). Renal levels of monocyte chemoattractant protein-1 (MCP-1) were determined using the mouse MCP-1 Quantikine ELISA kit (R&D Systems). All analyses were performed following the manufacturers’ instructions.

### 2.3. Histological Analysis and Immunohistochemical (IHC) Staining

Kidney tissues were fixed, dehydrated, and embedded in paraffin for periodic acid-Schiff (PAS) and Masson’s trichrome staining. The degree of tubular injury was scored based on the percentage of injured tubules: 0, 0%; 1, ≤10%; 2, 11–25%; 3, 26–45%; 4, 46–75%; and 5, 76–100% [23,24]. Tubular injury was analyzed in 10 random cortical fields (×400) per sample. For IHC staining, the sections were deparaffinized and rehydrated. After antigen retrieval, the sections were incubated with antibodies against α-smooth muscle actin (α-SMA; Sigma-Aldrich, St. Louis, MO, USA), 4-hydroxynonenal (4-HNE; Abcam, Cambridge, MA, USA), NADPH oxidase 4 (NOX-4; Novus Biologicals, Littleton, CO, USA) and F4/80 (Santa Cruz Biotechnology, Santa Cruz, CA, USA). Then, the sections were reacted with secondary antibodies. Slides were viewed and captured using a slide scanner (3DHISTECH Pannoramic MIDI, Budapest, Hungary). Quantification of positive staining for Masson’s trichrome, α-SMA, 4-HNE or NOX-4 was analyzed using the IMT i-Solution software (IMT i-Solution, Inc., Coquitlam, BC, Canada) in 10 random cortical fields (×400) per sample according to the manufacturer’s instructions. This computer-assisted automated image analyzer has been widely used to analyze positively stained areas [25,26,27]. The number of F4/80-postive cells was counted in 10 random cortical fields (×600) per sample.

### 2.4. Immunofluorescent (IF) Staining

Kidney sections were incubated with anti-8-hydroxy-2’-deoxyguanosine (8-OHdG) antibody (Santa Cruz Biotechnology) and anti-Ly6B.2 antibody (Abcam). After washing, the sections were probed with secondary antibodies conjugated with Alexa Fluor 647 or Alexa Fluor 594. To detect the brush border of proximal tubules, the FITC-labeled lotus tetragonolobus lectin (LTL; Vector Laboratories, Burlingame, CA, USA) was used. Nuclei were counterstained with DAPI. Images were viewed and captured using a confocal microscope (Nikon, Tokyo, Japan). Quantification of positive staining for LTL was analyzed using the IMT i-Solution software (IMT i-Solution, Inc.) in random cortical fields (×400) per sample according to the manufacturer’s instructions. The number of 8-OHdG-positive cells or Ly6B.2-positive cells was counted in 10 random cortical fields (×600) per sample.

### 2.5. Western Blotting

Total protein was extracted from tissues using a RIPA lysis buffer (Cayman Chemical). Protein samples (10 μg) were loaded onto precast gradient polyacrylamide gels (Bio-Rad Laboratories, Hercules, CA, USA). After electrophoresis, the separated proteins were transferred to nitrocellulose membranes. The, the membranes were reacted with antibodies against fibronectin (Abcam), TGF-β1 (R&D Systems), CTGF (Abcam), glyceraldehyde-3-phosphate dehydrogenase (GAPDH; Cell Signaling Technology, Danvers, MA, USA), vimentin (Cell Signaling Technology), α-SMA (Sigma-Aldrich), NOX-4 (Novus Biologicals), catalase (Abcam), manganese superoxide dismutase (MnSOD; Abcam), spliced X-box binding protein 1 (XBP1s; Cell Signaling Technology), eukaryotic initiation factor 2α (eIF2α; Cell Signaling Technology), p-eIF2α (Cell Signaling Technology), activating transcription factor 4 (ATF4; Cell Signaling Technology), ATF6 (Abcam), CCAAT/enhancer-binding protein homologous protein (CHOP; Thermo Fisher Scientific, Waltham, MA, USA), cleaved caspase-3 (Cell Signaling Technology), cleaved poly(ADP-ribose) polymerase-1 (cleaved PARP-1; Cell Signaling Technology), p53 (Cell Signaling Technology), Bax (Santa Cruz Biotechnology), receptor-interacting serine/threonine-protein kinase 1 (RIPK1; Cell Signaling Technology), RIPK3 (Cell Signaling Technology), mixed lineage kinase domain-like pseudokinase (MLKL; Cell Signaling Technology), inhibitor κB-α (IκB-α; Cell Signaling Technology), p-IκB-α (Cell Signaling Technology), nuclear factor-κB (NF-κB) p65 (Cell Signaling Technology), p-NF-κB p65 (Cell Signaling Technology) and intercellular adhesion molecule-1 (ICAM-1; Santa Cruz Biotechnology). Then, the membranes were incubated with secondary antibodies. The blots were visualized using the iBright CL1500 Imaging System (Thermo Fisher Scientific) and enhanced chemiluminescence reagents (Thermo Fisher Scientific). Relative protein levels were quantified with ImageJ software (NIH, USA) using GAPDH as an internal control.

### 2.6. Quantitative Real-Time Polymerase Chain Reaction (qRT-PCR)

Total RNA were extracted from tissues using TRIzol reagent (Sigma-Aldrich). The reverse transcription of extracted RNA was conducted for cDNA synthesis. Real-time PCR was performed using the specific primers (Table 1) in the Thermal Cycler Dice Real Time System III (TaKaRa, Tokyo, Japan). Relative expression was calculated by using the 2^−ΔΔCT^ method. All data were normalized to GAPDH.

### 2.7. Assessment of Oxidative Stress and Antioxidant Enzyme Activities

Malondialdehyde (MDA) and 8-OHdG levels were measured using the MDA assay kit (Sigma-Aldrich) and the 8-OHdG assay kit (Abcam), respectively. Reduced glutathione (GSH) and oxidized glutathione (GSSG) levels were determined using the GSH detection kit (Enzo Life Sciences, Farmingdale, NY, USA). Activities of catalase and SOD were measured using commercial kits (Invitrogen, Carlsbad, CA, USA). MPO activity was determined using the MPO activity assay kit (Abcam). All analyses were performed following the manufacturers’ instructions.

### 2.8. TdT-Mediated dUTP Nick End Labeling (TUNEL) Staining

Apoptosis were detected in tissues using a TUNEL assay kit (Roche Diagnostics, Indianapolis, IN, USA) following the manufacturer′s protocol. Briefly, the sections were deparaffinized, permeabilized, and incubated in the TUNEL reaction mixture. Images were viewed and captured using a confocal microscope (Nikon). Positive cells were counted in 10 random cortical fields (×600) per sample.

### 2.9. Statistical Analysis

Data were expressed as the mean ± SEM. Statistical significance was assessed by one-way analysis of variance (ANOVA) with Bonferroni’s multiple comparison tests. A *p*-value less than 0.05 was considered significant.

## 3. Results

### 3.1. CS Ameliorated Renal Dysfunction and Tubular Injury in UUO Mice

**Figure 1:**
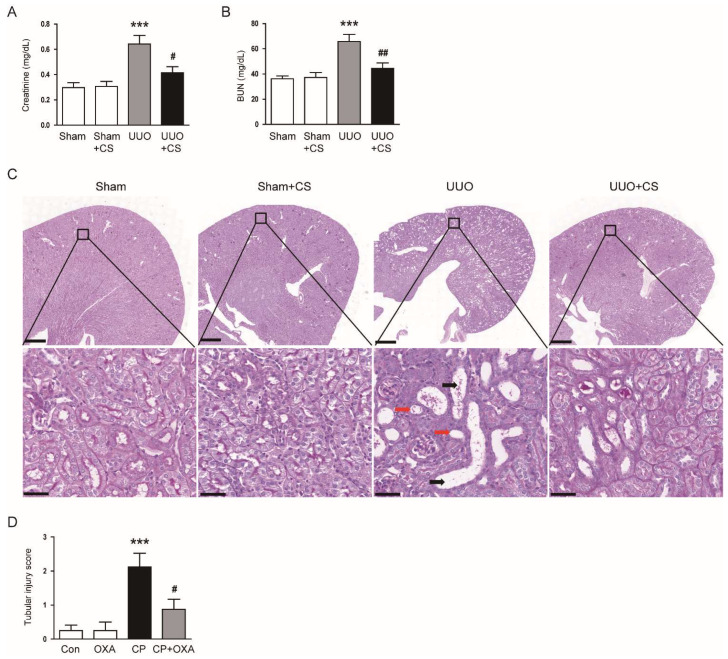
Effects of CS on renal dysfunction and histological abnormalities in UUO-operated mice. (**A**) Serum creatinine levels. (**B**) BUN levels. (**C**) PAS staining of kidney sections. Red arrows indicate tubular atrophy. Black arrows indicate tubular dilatation. Scale bars in the upper panel = 500 μm. Scale bars in the lower panel = 40 μm. (**D**) Tubular injury score. *** *p* < 0.001 vs. Sham. ^#^ *p* < 0.05 and ^##^ *p* < 0.01 vs. UUO.

To first evaluate the effect of CS on renal function in UUO mice, we measured serum levels of creatinine and BUN, established indicators of renal function [28,29], in each group. Serum levels of creatinine and BUN were increased after UUO surgery (Creatinine: Sham, 0.30 ± 0.04 mg/dL vs. UUO, 0.64 ± 0.07 mg/dL, *p* < 0.001; BUN: Sham, 36.3 ± 2.2 mg/dL vs. UUO, 65.8 ± 5.7 mg/dL, *p* < 0.001) (Figure 1A,B). CS treatment significantly reduced serum levels of both indicators in UUO mice (Creatinine: UUO, 0.64 ± 0.07 mg/dL vs. UUO+CS, 0.42 ± 0.05 mg/dL, *p* < 0.05; BUN: UUO, 65.8 ± 5.7 mg/dL vs. UUO+CS, 44.4 ± 4.4 mg/dL, *p* < 0.01) (Figure 1A,B). PAS staining showed that UUO mice developed tubular dilatation, tubular atrophy and infiltration of inflammatory cells (Figure 1C). CS attenuated these histological alterations in UUO mice, while CS alone had no effect on tubular morphology (Figure 1C). As a result of semi-quantitative analysis, the increased tubular injury score in UUO mice was significantly reduced by CS (UUO, 3.5 ± 0.3 vs. UUO+CS, 1.6 ± 0.3, *p* < 0.001) (Figure 1D).

**Figure 2:**
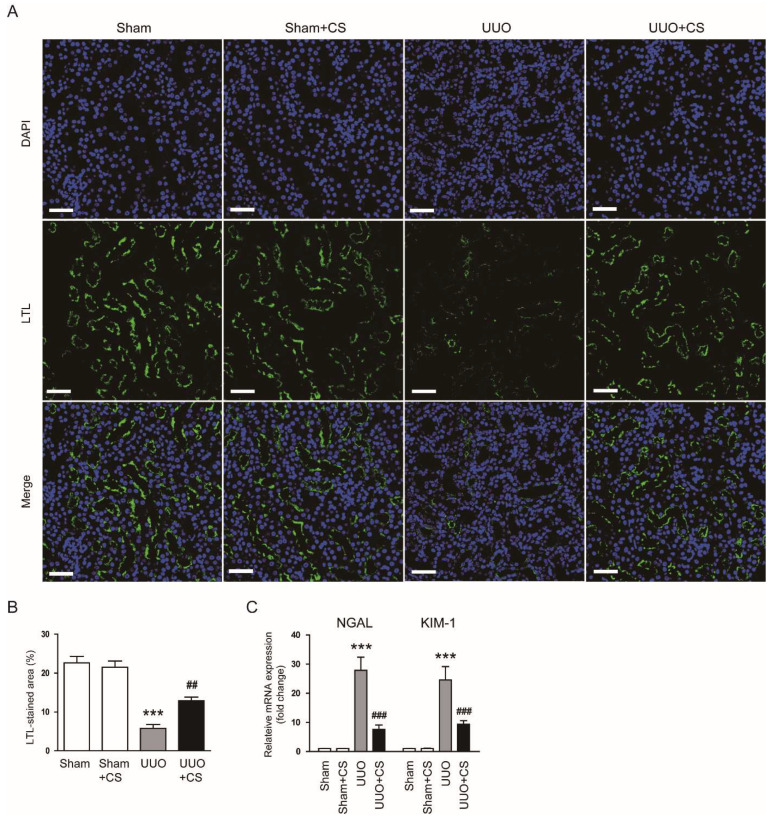
Effects of CS on loss of proximal tubule brush border and expression of tubular injury markers in UUO mice. (**A**) IF staining of kidney sections for LTL. Scale bar = 50 μm. (**B**) Percentages of LTL-stained area. (**C**) Renal *NGAL* and *KIM-1* mRNA levels. *** *p* < 0.001 vs. Sham. ^##^ *p* < 0.01 and ^###^ *p* < 0.001 vs. UUO.

Staining with LTL, a specific marker for the brush border of proximal tubules [30,31], showed a higher percentage of LTL-stained area in the UUO group than in the Sham group (Sham, 22.6 ± 1.7 % vs. UUO, 5.8 ± 1.0 %, *p* < 0.001) (Figure 2A,B). However, UUO-induced loss of the proximal tubule brush border was significantly attenuated by CS (UUO, 5.8 ± 1.0 % vs. UUO+CS, 12.9 ± 1.0 %, *p* < 0.01) (Figure 2A,B). Furthermore, CS treatment reduced mRNA expression of neutrophil gelatinase-associated lipocalin (*NGAL*) and kidney injury molecule-1 (*KIM-1*), tubular injury markers [32,33], in kidneys of UUO mice (*NGAL*: UUO, 27.9 ± 4.5 vs. UUO+CS, 7.6 ± 1.5, *p* < 0.001; *KIM-1*: UUO, 24.6 ± 4.6 vs. UUO+CS, 9.4 ± 1.3, *p* < 0.001) (Figure 2C).

### 3.2. CS Alleviated Renal Fibrosis in UUO Mice

**Figure 3:**
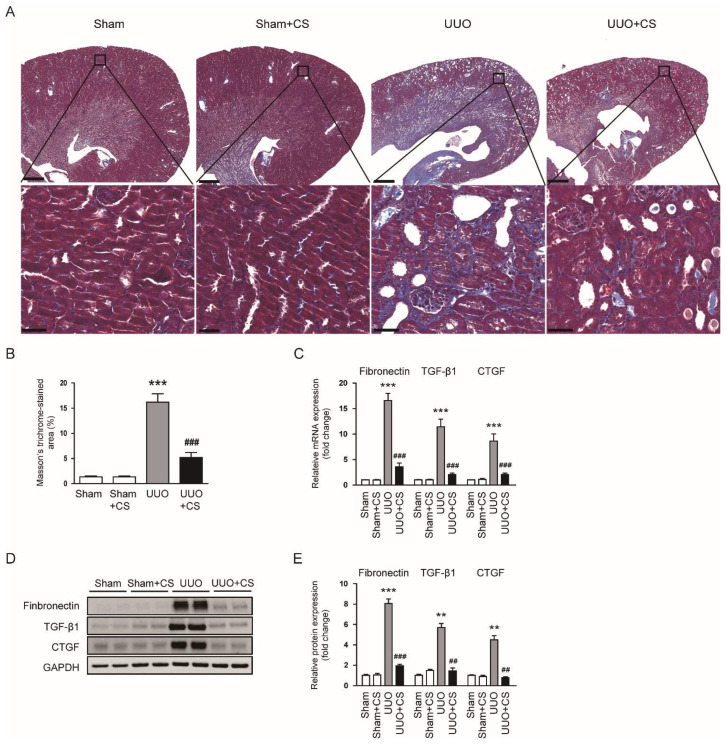
Effects of CS on renal fibrosis in UUO mice. (**A**) Masson’s trichrome staining of kidney sections. Scale bars in the upper panel = 500 μm. Scale bars in the lower panel = 40 μm. (**B**) Percentages of Masson’s trichrome-stained area. (**C**) Renal *fibronectin*, *TGF-β1* and *CTGF* mRNA levels. (**D**) Western blotting of fibronectin, TGF-β1 and CTGF. (**E**) Quantification of Western blots for fibronectin, TGF-β1 and CTGF. ** *p* < 0.01 and *** *p* < 0.001 vs. Sham. ^##^ *p* < 0.01 and ^###^ *p* < 0.001 vs. UUO.

Masson’s trichrome staining showed that the area with positive staining for collagen was increased after UUO surgery (Sham, 1.3 ± 0.2 % vs. UUO, 16.2 ± 1.7 %, *p* < 0.001) and CS remarkably decreased the fibrotic area (UUO, 16.2 ± 1.7 % vs. UUO+CS, 5.2 ± 1.0, *p* < 0.001) (Figure 3A,B). In addition, renal mRNA levels of *fibronectin*, *TGF-β1* and *CTGF* were reduced by CS (*fibronectin*: UUO, 16.6 ± 1.4 vs. UUO+CS, 3.6 ± 0.7, *p* < 0.001; *TGF-β1*: UUO, 11.4 ± 1.5 vs. UUO+CS, 2.0 ± 0.3, *p* < 0.001; *CTGF*: UUO, 8.6 ± 1.4 vs. UUO+CS, 2.0 ± 0.3, *p* < 0.001) (Figure 3C). These results were confirmed by the results of Western blotting (fibronectin: UUO, 8.1 ± 0.5 vs. UUO+CS, 2.0 ± 0.2, *p* < 0.001; TGF-β1: UUO, 5.7 ± 0.4 vs. UUO+CS, 1.5 ± 0.3, *p* < 0.01; CTGF: UUO, 4.5 ± 0.4 vs. UUO+CS, 0.8 ± 0.1, *p* < 0.01) (Figure 3D,E).

**Figure 4:**
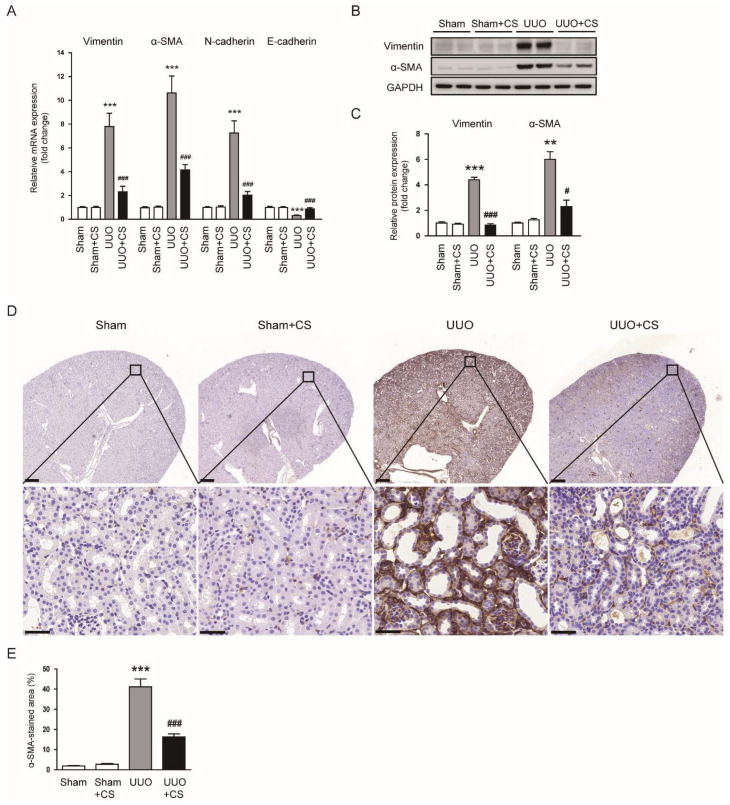
Effects of CS on myofibroblast accumulation in UUO mice. (**A**) Renal *vimentin*, *α-SMA*, *N-cadherin* and *E-cadherin* mRNA levels. (**B**) Western blotting of vimentin and α-SMA. (**C**) Quantification of Western blots for vimentin and α-SMA. (**D**) IHC staining of kidney sections for α-SMA. Scale bars in the upper panel = 500 μm. Scale bars in the lower panel = 40 μm. (**E**) Percentages of α-SMA-stained area. ** *p* < 0.01 and *** *p* < 0.001 vs. Sham. ^#^ *p* < 0.05 and ^###^ *p* < 0.001 vs. UUO.

CS treatment reduced renal mRNA levels of *vimentin*, *α-SMA* and *N-cadherin* while decreasing *E-cadherin* mRNA expression in UUO mice (*vimentin*: UUO, 7.8 ± 1.1 vs. UUO+CS, 2.3 ± 0.5, *p* < 0.001; *α-SMA*: UUO, 10.6 ± 1.4 vs. UUO+CS, 4.2 ± 0.4, *p* < 0.001; *N-cadherin*: UUO, 7.3 ± 1.0 vs. UUO+CS, 2.0 ± 0.3, *p* < 0.001; *E-cadherin*: UUO, 0.31 ± 0.05 vs. UUO+CS, 0.87± 0.11, *p* < 0.001) (Figure 4A). Protein levels of vimentin and α-SMA were also decreased by CS (vimentin: UUO, 4.4 ± 0.2 vs. UUO+CS, 0.9 ± 0.1, *p* < 0.001; α-SMA: UUO, 6.0 ± 0.6 vs. UUO+CS, 2.3 ± 0.5, *p* < 0.05) (Figure 4B,C). IHC staining confirmed the inhibitory effect of CS on α-SMA expression in kidneys of UUO mice (UUO, 41.2 ± 3.9 % vs. UUO+CS, 16.3 ± 1.5 %, *p* < 0.001) (Figure 4D,E).

### 3.3. CS Alleviated Oxidative Damage in UUO Mice

**Figure 5:**
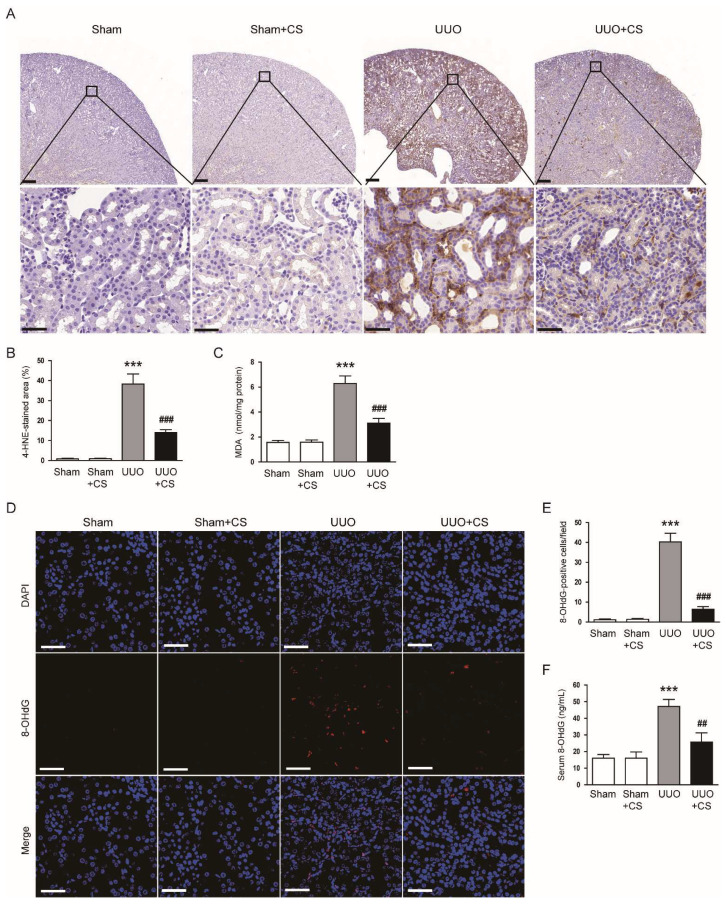
Effects of CS on oxidative stress in UUO mice. (**A**) IHC staining of kidney sections for 4-HNE. Scale bars in the upper panel = 200 μm. Scale bars in the lower panel = 40 μm. (**B**) Percentages of 4-HNE-stained area. (**C**) Renal MDA levels. (**D**) IF staining of kidney sections for 8-OHdG. Scale bar = 50 μm. (**E**) Number of 8-OHdG-positive cells per field. (**F**) Serum 8-OHdG levels. *** *p* < 0.001 vs. Sham. ^##^ *p* < 0.01 and ^###^ *p* < 0.001 vs. UUO.

Oxidative stress is a key contributor to renal fibrosis [6]. Thus, we evaluate the effect of CS on UUO-induced oxidative stress to explore the potential mechanism of action of CS. IHC staining for the lipid peroxidation product 4-HNE [34,35] revealed that the percentage of 4-HNE-stained area was higher in the UUO group than in in the Sham group (Sham, 0.8 ± 0.2 % vs. UUO, 38.3 ± 5.0 %, *p* < 0.001) (Figure 5A,B). CS treatment significantly decreased the area of 4-HNE staining in kidneys of UUO mice (UUO, 38.3 ± 5.0 % vs. UUO+CS, 14.0 ± 1.4 %, *p* < 0.001) (Figure 5A,B). Renal amount of the lipid peroxidation product MDA [34,35] was also reduced by CS (UUO, 6.3 ± 0.6 nmol/mg protein vs. UUO+CS, 3.1 ± 0.4 nmol/mg protein, *p* < 0.001) (Figure 5C). In addition, IF staining for 8-OHdG, an oxidative nucleoside product [36], revealed that the number of 8-OHdG-positive cells increased after UUO surgery (Sham, 1.1 ± 0.4 vs. UUO, 40.3 ± 4.4, *p* < 0.001) (Figure 5D,E). However, CS treatment remarkably reduced the number of 8-OHdG-positive cells (UUO, 40.3 ± 4.4 vs. UUO+CS, 6.4 ± 1.3, *p* < 0.001) (Figure 5D,E). Serum 8-OHdG levels were also decreased by CS (UUO, 47.1 ± 4.3 ng/mL vs. UUO+CS, 25.8 ± 5.4 ng/mL, *p* < 0.01) (Figure 5F).

**Figure 6:**
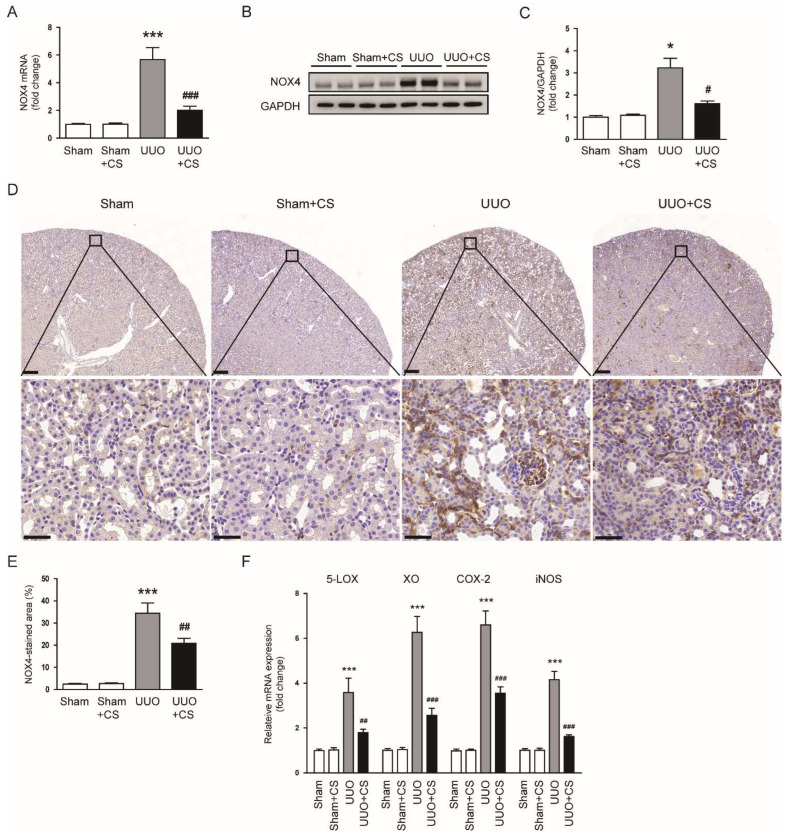
Effects of CS on pro-oxidant enzymes in UUO mice. (**A**) Renal NOX4 mRNA levels. (**B**) Western blotting of NOX4. (**C**) Quantification of Western blots for NOX4. (**D**) IHC staining of kidney sections for NOX4. Scale bars in the upper panel = 200 μm. Scale bars in the lower panel = 40 μm. (**E**) Percentages of NOX4-stained area. (**F**) Renal *5-LOX*, *XO*, *COX-2* and *iNOS* mRNA levels. * *p* < 0.05 and *** *p* < 0.001 vs. Sham. ^#^ *p* < 0.05, ^##^ *p* < 0.01 and ^###^ *p* < 0.001 vs. UUO.

To elucidate the mechanism by which CS inhibits UUO-induced oxidative damage, we first examined the expression of the pro-oxidant enzyme NOX4 in each group. The UUO group showed increased levels of *NOX4* mRNA and protein in kidneys compared to the Sham group (*NOX4* mRNA: Sham, 1.0 ± 0.1 vs. UUO, 5.7 ± 0.9, *p* < 0.001; NOX4 protein: Sham, 1.0 ± 0.1 vs. UUO, 3.2 ± 0.4, *p* < 0.05) (Figure 6A–C). CS treatment remarkable reduced UUO-induced *NOX4* mRNA and protein expression (*NOX4* mRNA: UUO, 5.7 ± 0.9 vs. UUO+CS, 2.0 ± 0.3, *p* < 0.001; NOX4 protein: UUO, 3.2 ± 0.4 vs. UUO, 1.6 ± 0.1, *p* < 0.05) (Figure 6A–C). IHC staining confirmed the inhibitory effect of CS on NOX4 expression in UUO mice (UUO, 34.5 ± 4.6 % vs. UUO+CS, 20.9 ± 2.2 %, *p* < 0.01) (Figure 6D,E). Renal mRNA levels of 5-lipoxygenase (*5-LOX*), xanthine oxidase (*XO*), cyclooxygenase-2 (*COX-2*) and inducible nitric oxide synthase (*iNOS*) were also reduced by CS (*5-LOX*: UUO, 3.6 ± 0.6 vs. UUO+CS, 1.8 ± 0.2, *p* < 0.01; *XO*: UUO, 6.3 ± 0.7 vs. UUO+CS, 2.6 ± 0.3, *p* < 0.001; *COX-2*: UUO, 6.6 ± 0.6 vs. UUO+CS, 3.5 ± 0.3, *p* < 0.001; *iNOS*: UUO, 4.2 ± 0.4 vs. UUO+CS, 1.6 ± 0.1, *p* < 0.001) (Figure 6F).

**Figure 7:**
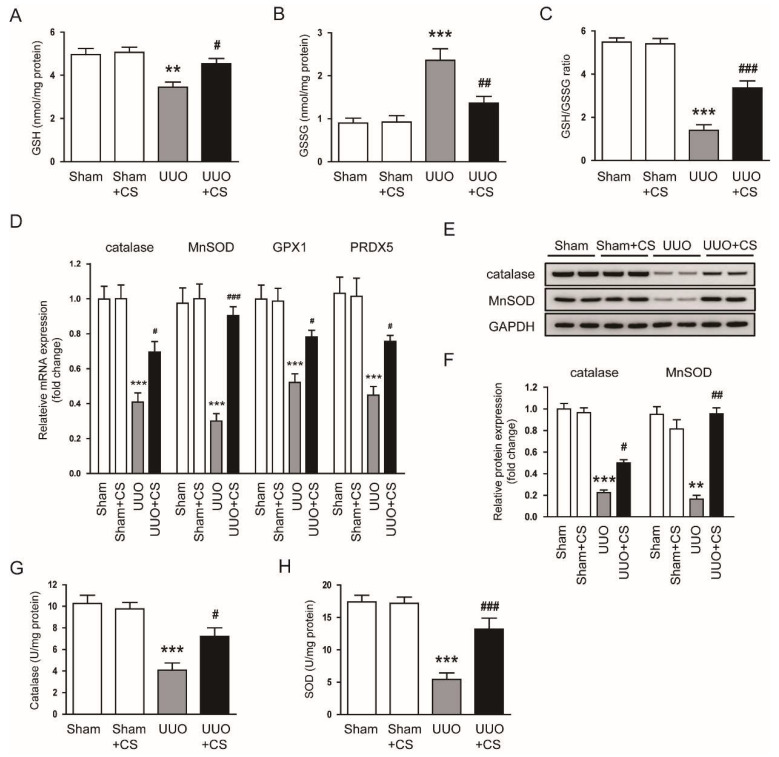
Effects of CS on antioxidant enzymes in UUO mice. (**A**) Renal GSH levels. (**B**) Renal GSSG levels. (**C**) GSH/GSSG ratio. (**D**) Renal *catalase*, *MnSOD*, *GPX1* and *PRDX5* mRNA levels. (**E**) Western blotting of catalase and MnSOD. (**F**) Quantification of Western blots for catalase and MnSOD. (**G**) Catalase activities in kidney tissues. (**H**) SOD activities in kidney tissues. ** *p* < 0.01 and *** *p* < 0.001 vs. Sham. ^#^ *p* < 0.05, ^##^ *p* < 0.01 and ^###^ *p* < 0.001 vs. UUO.

In addition, CS treatment significantly increased renal levels of GSH, a major endogenous antioxidant [37], and decreased renal GSSG levels, thereby increasing the GSH/GSSG ratio in UUO mice (GSH: UUO, 3.5 ± 0.2 nmol/mg protein vs. UUO+CS, 4.5 ± 0.2 nmol/mg protein, *p* < 0.05; GSSG: UUO, 2.4 ± 0.3 nmol/mg protein vs. UUO+CS, 1.4 ± 0.2 nmol/mg protein, *p* < 0.01; GSH/GSSG: UUO, 1.4 ± 0.3 vs. UUO+CS, 3.4 ± 0.3, *p* < 0.001) (Figure 7A–C). CS reduced renal mRNA expression of the antioxidant enzymes *catalase*, *MnSOD*, glutathione peroxidase 1 (*GPX1*) and peroxiredoxin-5 (*PRDX5*) (*catalase*: UUO, 0.41 ± 0.05 vs. UUO+CS, 0.70 ± 0.06, *p* < 0.05; *MnSOD*: UUO, 0.30 ± 0.04 vs. UUO+CS, 0.90 ± 0.05, *p* < 0.001; *GPX1*: UUO, 0.52 ± 0.05 vs. UUO+CS, 0.78 ± 0.04, *p* < 0.05; *PRDX5*: UUO, 0.45 ± 0.05 vs. UUO+CS, 0.76 ± 0.03, *p* < 0.05) (Figure 7D). Reduced renal protein expression of catalase and MnSOD in UUO mice was also increased by CS (catalase: UUO, 0.23 ± 0.03 vs. UUO+CS, 0.50 ± 0.03, *p* < 0.05; MnSOD: UUO, 0.17 ± 0.04 vs. UUO+CS, 0.96 ± 0.06, *p* < 0.01) (Figure 7E,F). Furthermore, CS treatment increased enzymatic activities of catalase and SOD in kidneys of UUO mice (catalase: UUO, 4.1 ± 0.7 U/mg protein vs. UUO+CS, 7.2 ± 0.8 U/mg protein, *p* < 0.05; MnSOD: UUO, 5.4 ± 1.0 U/mg protein vs. UUO+CS, 13.2 ± 1.7 U/mg protein, *p* < 0.001) (Figure 7G,H).

### 3.4. CS Suppressed Endoplasmic Reticulum Stress (ER Stress) in UUO Mice

**Figure 8:**
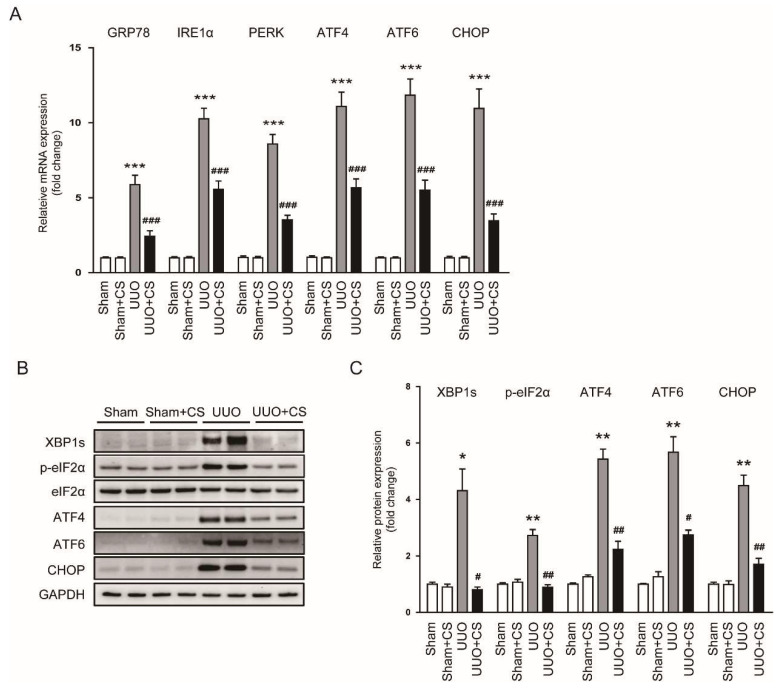
Effects of CS on ER stress in UUO mice. (**A**) Renal *GRP78*, *IRE1α*, *PERK*, *ATF4*, *ATF6* and *CHOP* mRNA levels. (**B**) Western blotting of XBP1s, p-eIF2α, ATF4, ATF6 and CHOP. (**C**) Quantification of Western blots for XBP1s, p-eIF2α, ATF4, ATF6 and CHOP. * *p* < 0.05, ** *p* < 0.01 and *** *p* < 0.001 vs. Sham. ^#^ *p* < 0.05, ^##^ *p* < 0.01 and ^###^ *p* < 0.001 vs. UUO.

Oxidative stress can induce ER stress, leading to cell death and inflammation [38,39]. ER stress is known to play an important role in the pathogenesis of CKD [40,41]. Therefore, we next examined the effect of CS on ER stress in UUO mice. Renal mRNA levels of glucose-regulated protein 78 (*GRP78*), inositol-requiring enzyme 1α (*IRE1α*), protein kinase RNA-like ER kinase (*PERK*), *ATF4*, *ATF6* and *CHOP* were increased after UUO surgery (*GRP78*: Sham, 1.00 ± 0.06 vs. UUO, 5.9 ± 0.6, *p* < 0.001; *IRE1α*: Sham, 1.00 ± 0.07 vs. UUO, 10.3 ± 0.7, *p* < 0.001; *PERK*: Sham, 1.00 ± 0.11 vs. UUO, 8.6 ± 0.6, *p* < 0.001; *ATF4*: Sham, 1.00 ± 0.10 vs. UUO, 11.1 ± 1.0, *p* < 0.001; *ATF6*: Sham, 1.00 ± 0.06 vs. UUO, 11.8 ± 1.1, *p* < 0.001; *CHOP*: Sham, 1.00 ± 0.09 vs. UUO, 11.0 ± 1.3, *p* < 0.001) (Figure 8A). CS treatment remarkably downregulated renal expression of the ER stress markers (*GRP78*: UUO, 5.9 ± 0.6 vs. UUO+CS, 2.4 ± 0.4, *p* < 0.001; *IRE1α*: UUO, 10.3 ± 0.7 vs. UUO+CS, 5.6 ± 0.6, *p* < 0.001; *PERK*: UUO, 8.6 ± 0.6 vs. UUO+CS, 3.5 ± 0.3, *p* < 0.001; *ATF4*: UUO, 11.1 ± 1.0 vs. UUO+CS, 5.7 ± 0.6, *p* < 0.001; *ATF6*: UUO, 11.8 ± 1.1 vs. UUO+CS, 5.5 ± 0.7, *p* < 0.001; *CHOP*: UUO, 11.0 ± 1.3 vs. UUO+CS, 3.5 ± 0.5, *p* < 0.001) (Figure 8A). Protein expression of XBP1s, p-eIF2α, ATF4, ATF6 and CHOP was also reduced by CS (XBP1s: UUO, 4.3 ± 0.8 vs. UUO+CS, 0.8 ± 0.1, *p* < 0.05; p-eIF2α: UUO, 2.7 ± 0.2 vs. UUO+CS, 0.9 ± 0.1, *p* < 0.01; ATF4: UUO, 5.4 ± 0.4 vs. UUO+CS, 2.2 ± 0.3, *p* < 0.001; ATF6: UUO, 5.7 ± 0.6 vs. UUO+CS, 2.7 ± 0.2, *p* < 0.05; CHOP: UUO, 4.5 ± 0.4 vs. UUO+CS, 1.7 ± 0.2, *p* < 0.01) (Figure 8B,C).

### 3.5. CS Inhibited Tubular Cell Death in UUO Mice

**Figure 9:**
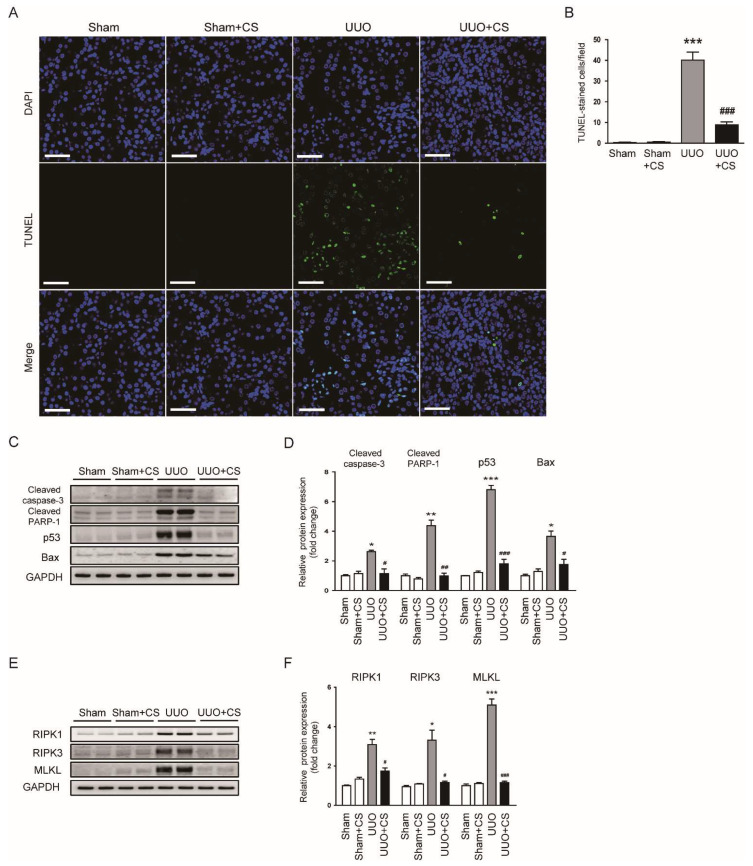
Effects of CS on tubular cell death in UUO mice. (**A**) TUNEL staining on kidney sections. Scale bar = 50 μm. (**B**) Number of TUNEL-positive cells. (**C**) Western blotting of cleaved caspase-3, cleaved PARP-1, p53 and Bax. (**D**) Quantification of protein expression of cleaved caspase-3, cleaved PARP-1, p53 and Bax. (**E**) Western blotting of RIPK1, RIPK3 and MLKL. (**F**) Quantification of protein expression of RIPK1, RIPK3 and MLKL. * *p* < 0.05, ** *p* < 0.01 and *** *p* < 0.001 vs. Sham. ^#^ *p* < 0.05, ^##^ *p* < 0.01 and ^###^ *p* < 0.001 vs. UUO.

Tubular cell death also plays an important role in renal fibrosis [42,43]. Emerging evidence highlights the pathogenic role of apoptosis and necroptosis in the pathogenesis of renal fibrosis [44,45]. TUNEL staining on kidney sections was performed to detect apoptotic cells. UUO mice displayed increased number of TUNEL-stained cells in kidneys (Sham, 0.4 ± 0.2 vs. UUO, 40.1 ± 3.9, *p* < 0.001) (Figure 9A,B). CS treatment significantly inhibited UUO-induced renal cell apoptosis (UUO, 40.1 ± 3.9 vs. UUO+CS, 8.9 ± 1.4, *p* < 0.001) (Figure 9A,B). Increased protein levels of cleaved caspase-3, cleaved PARP-1, p53 and Bax after UUO surgery were also decreased by CS (cleaved caspase-3: UUO, 2.6 ± 0.1 vs. UUO+CS, 1.1 ± 0.3, *p* < 0.05; cleaved PARP-1: UUO, 4.4 ± 0.4 vs. UUO+CS, 1.0 ± 0.2, *p* < 0.01; p53: UUO, 6.8 ± 0.3 vs. UUO+CS, 1.8 ± 0.3, *p* < 0.001; Bax: UUO, 3.6 ± 0.4 vs. UUO+CS, 1.8 ± 0.4, *p* < 0.05) (Figure 9C,D). Furthermore, CS treatment remarkably reduced protein expression of RIPK1, RIPK3 and MLKL (RIPK1: UUO, 3.1 ± 0.3 vs. UUO+CS, 1.7 ± 0.2, *p* < 0.05; RIPK3: UUO, 3.3 ± 0.5 vs. UUO+CS, 1.2 ± 0.1, *p* < 0.05; MLKL: UUO, 5.1 ± 0.3 vs. UUO+CS, 1.2 ± 0.1, *p* < 0.001) (Figure 9E,F).

### 3.6. CS Attenuated Inflammatory Responses in UUO Mice

**Figure 10:**
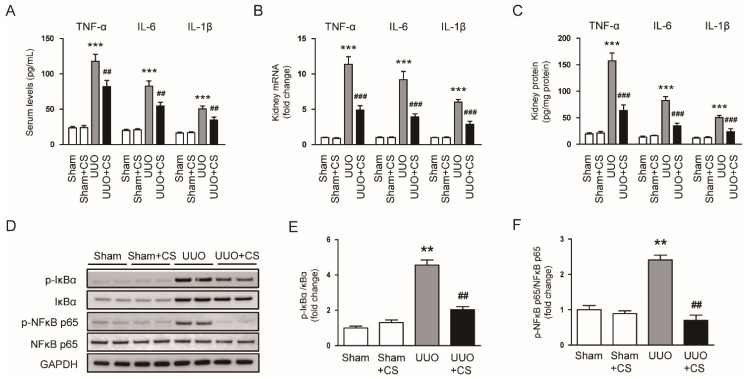
Effects of CS on cytokine production in UUO mice. (**A**) Serum TNF-α, IL-6 and IL-1β levels. (**B**) Renal *TNF-α*, *IL-6* and *IL-1β* mRNA levels. (**C**) Renal TNF-α, IL-6 and IL-1β protein levels. (**D**) Western blotting of p-IκB-α and p-NF*κ*B p65. (**E**) Quantification of Western blots for p-IκB-α. (**F**) Quantification of Western blots for p-NF*κ*B p65. ** *p* < 0.01 and *** *p* < 0.001 vs. Sham. ^##^ *p* < 0.01 and ^###^ *p* < 0.001 vs. UUO.

Severe and prolonged inflammation can promote renal fibrosis [46]. UUO mice exhibited increased serum levels of TNFα, IL-6 and IL-1β (TNF-α: Sham, 23.4 ± 2.2 pg/mL vs. UUO, 117.8 ± 10.0 pg/mL, *p* < 0.001; IL-6: Sham, 19.9 ± 1.6 pg/mL vs. UUO, 82.6 ± 7.3 pg/mL, *p* < 0.001; IL-1β: Sham, 16.1 ± 1.6 pg/mL vs. UUO, 50.4 ± 4.1 pg/mL, *p* < 0.001) (Figure 10A). CS treatment reduced serum levels of these pro-inflammatory cytokines (TNF-α: UUO, 117.8 ± 10.0 pg/mL vs. UUO+CS, 81.9 ± 8.8 pg/mL, *p* < 0.01; IL-6: UUO, 82.6 ± 7.3 pg/mL vs. UUO+CS, 54.5 ± 5.3 pg/mL, *p* < 0.01; IL-1β: UUO, 50.4 ± 4.1 pg/mL vs. UUO+CS, 34.5 ± 4.3 pg/mL, *p* < 0.01) (Figure 10A). Increased renal mRNA expression of *TNFα*, *IL-6* and *IL-1β* was also significantly decreased by CS (*TNF-α*: UUO, 11.4 ± 1.1 vs. UUO+CS, 4.9 ± 0.6, *p* < 0.001; *IL-6*: UUO, 9.2 ± 1.2 vs. UUO+CS, 3.9 ± 0.5, *p* < 0.001; *IL-1β*: UUO, 6.0 ± 0.4 vs. UUO+CS, 2.9 ± 0.5, *p* < 0.001) (Figure 10B). Quantitative measurement of TNFα, IL-6 and IL-1β proteins in kidneys also confirmed the inhibitory effect of CS on cytokine production (TNF-α: UUO, 157.6 ± 14.4 pg/mg protein vs. UUO+CS, 63.5 ± 10.9 pg/mg protein, *p* < 0.001; IL-6: UUO, 82.6 ± 7.3 pg/mg protein vs. UUO+CS, 34.5 ± 5.3 pg/mg protein, *p* < 0.001; IL-1β: UUO, 50.4 ± 4.1 pg/mg protein vs. UUO+CS, 23.8 ± 5.4 pg/mg protein, *p* < 0.001) (Figure 10C). Phosphorylation of IκBα and NFκB p65 proteins was increased in the UUO mice than in the Sham group (p-IκBα: Sham, 1.0 ± 0.1 vs. UUO, 4.6 ± 0.3, *p* < 0.01; p-NFκB p65: Sham, 1.0 ± 0.1 vs. UUO, 2.4 ± 0.1, *p* < 0.01) (Figure 10D,E). CS remarkably inhibited UUO-induced phosphorylation of IκBα and NFκB p65 (p-IκBα: Sham, UUO, 4.6 ± 0.3 vs. UUO+CS, 2.0 ± 0.2, *p* < 0.01; p-NFκB p65: UUO, 2.4 ± 0.1 vs. UUO+CS, 0.7 ± 0.2, *p* < 0.01) (Figure 10D,E).

**Figure 11:**
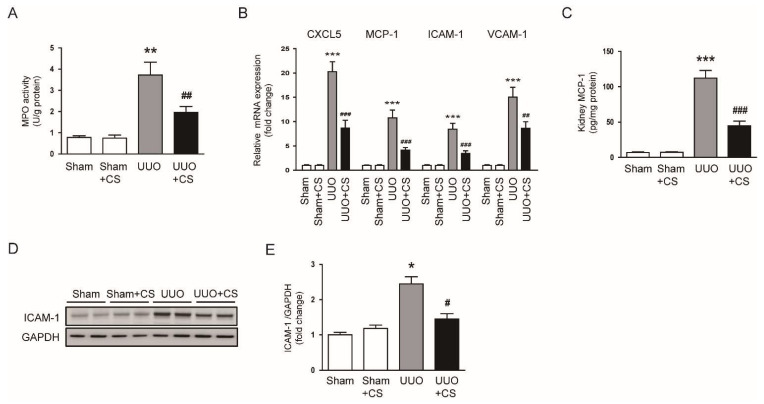
Effects of CS on expression of chemokines and adhesion molecules in UUO mice. (**A**) Renal MPO activities. (**B**) Renal *CXCL5*, *MCP-1*, *ICAM-1* and *VCAM-1* mRNA levels. (**C**) Renal MCP-1 protein levels. (**D**) Western blotting of ICAM-1. (**E**) Quantification of Western blots for ICAM-1. * *p* < 0.05, ** *p* < 0.01 and *** *p* < 0.001 vs. Sham. ^#^ *p* < 0.05, ^##^ *p* < 0.01 and ^###^ *p* < 0.001 vs. UUO.

Previous studies have shown that immune cells such as neutrophils and macrophages contribute to the development and progression of renal fibrosis [46]. We measured renal activity of MPO, an enzyme secreted by activated neutrophils and macrophages, in each group. Renal MPO activity was largely increased in the UUO group compared to the Sham group (Sham, 0.8 ± 0.1 U/g protein vs. UUO, 3.7 ± 0.6 U/g protein, *p* < 0.01) (Figure 11A). Increased activity of MPO was remarkably inhibited by CS (UUO, 3.7 ± 0.6 U/g protein vs. UUO+CS, 2.0 ± 0.3 U/g protein, *p* < 0.01) (Figure 11A). CS treatment reduced mRNA levels of C-X-C motif chemokine ligand 5 (*CXCL5*), *MCP-1*, *ICAM-1*, vascular cell adhesion protein 1 (*VCAM-1*) in kidneys of UUO mice (*CXCL5*: UUO, 20.3 ± 2.0 vs. UUO+CS, 8.7 ± 1.6, *p* < 0.001; *MCP-1*: UUO, 10.7 ± 1.6 vs. UUO+CS, 4.1 ± 0.5, *p* < 0.001; *ICAM-1*: UUO, 8.4 ± 1.2 vs. UUO+CS, 3.5 ± 0.5, *p* < 0.001; *VCAM-1*: UUO, 15.1 ± 2.0 vs. UUO+CS, 8.6 ± 1.4, *p* < 0.01) (Figure 11B). Renal levels of MCP-1 protein were reduced by CS (UUO, 112.1 ± 10.9 pg/mg protein vs. UUO+CS, 44.6 ± 6.8 pg/mg protein, *p* < 0.001) (Figure 11C). Western blot analysis showed that CS also decreased ICAM-1 protein expression in kidneys of UUO mice (UUO, 2.5 ± 0.2 vs. UUO+CS, 1.5 ± 0.2, *p* < 0.05) (Figure 11D,E).

**Figure 12:**
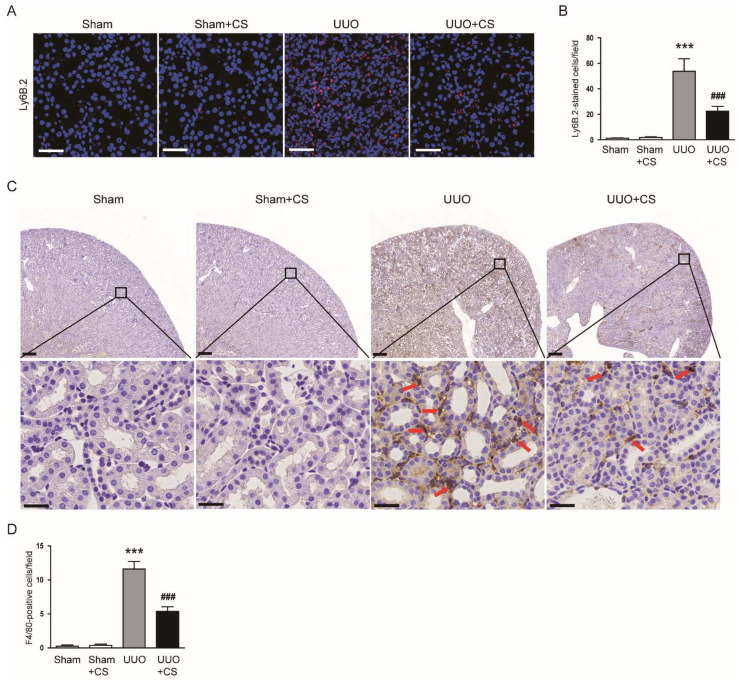
Effects of CS on immune cell infiltration in UUO mice. (**A**) IF staining of kidney sections for Ly6B.2. Scale bar = 50 μm. (**B**) Number of Ly6B.2-positive cells per field. (**C**) IHC staining of kidney sections for F4/80. Red arrows indicate positively stained cells. Scale bars in the upper panel = 200 μm. Scale bars in the lower panel = 60 μm. (**D**) Number of F4/80-positive cells per field. *** *p* < 0.001 vs. Sham. ^###^ *p* < 0.001 vs. UUO.

IF staining for Ly6B.2, a neutrophil marker [47], showed that the number of Ly6B.2-stained cells was decreased by CS (UUO, 53.8 ± 9.9 vs. UUO+CS, 22.5 ± 3.9, *p* < 0.001) (Figure 12A,B). CS treatment also reduced macrophage accumulation as indicated by a decrease in cells stained with the macrophage marker F4/80 [48] (UUO, 11.6 ± 1.1 vs. UUO+CS, 5.4 ± 0.7, *p* < 0.001) (Figure 12C,D).

## 4. Discussion

Rosemary is an herb that has long been used as an anti-inflammatory and analgesic agent [49]. Recent studies have shown that rosemary also has anti-tumor and anti-diabetic properties [50]. In addition, rosemary is considered an important source of natural antioxidants [51]. The diterpenes CS and carnosic acid are two of the most abundant bioactive compounds found in rosemary. These compounds contribute up to 90% of the rosemary’s antioxidant potential [49] and exert beneficial effects on various inflammatory diseases [13,52]. Carnosic acid has been shown to have a protective effect on acute kidney injury induced by lipopolysaccharide (LPS), cisplatin [53] or cadmium [54]. Carnosic acid also attenuated UUO-induced renal fibrosis [55] and streptozotocin-induced diabetic nephropathy [56]. On the other hand, CS treatment alleviated ischemia/reperfusion-induced acute kidney injury [20]. However, whether CS has a beneficial effect on renal injury and fibrosis induced by UUO has not yet been investigated. In this study, we found that CS improved renal function in UUO mice, as reflected by a decrease in serum creatinine and BUN levels. UUO mice displayed histopathological features of CKD such as tubular dilatation, tubular atrophy, inflammatory cell infiltration, loss of the brush border of proximal tubules, and interstitial fibrosis. These structural abnormalities were remarkably attenuated by CS. Increased expression of the tubular injury markers *NGAL* and *KIM-1* was also reduced by CS. Collectively, these data suggest that CS has a protective effect on UUO-induced renal injury and fibrosis. In this study, we also found using qRT-PCR, Western blot analysis and IHC staining that CS treatment decreased the renal expression of TGF-β1, CTGF, vimentin, α-SMA and N-cadherin while increasing E-cadherin. Renal fibrosis is characterized by excessive deposition of ECM and is the final common outcome of CKD [4]. Differentiated myofibroblasts during fibrosis are the main source of ECM and express the mesenchymal markers vimentin, α-SMA and N-cadherin with loss of epithelial markers such as E-cadherin [5]. TGF-β has been known to play an important role in myofibroblast differentiation and activation [57,58]. The TGF-β family consists of three isoforms: TGF-β1, TGF-β2 and TGF-β3. Among them, TGF-β1 is considered as a key mediator in renal fibrosis [57]. TGF-β1 induces the differentiation of fibroblasts into myofibroblasts and activates myofibroblasts to produce ECM proteins [58]. CTGF is also an important regulator of renal fibrosis and potentiates the TGF-β signaling pathway [59].

In this study, we observed increased oxidative stress in injured kidneys of UUO mice, as evidenced by an increase in 4-HNE stained area, MDA levels and the number of 8-OHdG-positive cells. Consistent with our findings, previous studies also showed that UUO mice had increased oxidative stress [60,61]. Interestingly, CS treatment remarkably attenuated UUO-induced oxidative stress. Our data support the notion that CS has a potent antioxidant activity [49]. Kalantar et al. showed that CS treatment ameliorated bleomycin-induced lung injury by inhibiting oxidative stress [62]. Saeed et al. reported that CS attenuated chronic stress-related brain damage through its antioxidant effect [63]. Therefore, the antioxidant action of CS may be a main contributor to its protective effect on UUO-induced renal injury and fibrosis. An imbalance between pro-oxidant and antioxidant systems has been shown to cause oxidative stress, contributing to renal fibrosis [6]. NOX4 is a main source of ROS in the kidney and plays an important role in renal fibrosis [64]. Previous studies have reported NOX4 upregulation in the UUO model [65,66]. Reduced expression of NOX4 contributed to the improvement of renal interstitial fibrosis in the UUO model [67,68]. In this study, we observed that CS treatment reduced *NOX4* mRNA and protein expression in kidneys of UUO mice. Other pro-oxidant enzymes including *5-LOX*, *XO*, *COX-2* and *iNOS* were also downregulated by CS. These results indicate that CS inhibited UUO-induced oxidative injury through suppressing pro-oxidant enzymes. We also found that UUO surgery induced depletion of the endogenous antioxidant GSH and downregulation of *catalase*, *MnSOD*, *GPX1* and *PRDX5* in the kidney. Depletion of GSH and decreased expression of the antioxidant enzymes was markedly reversed by CS. CS treatment also increased the activity of catalase and SOD in kidneys of UUO mice. Previous studies have shown that besides upregulation of pro-oxidant enzymes, UUO mice displayed decreased renal expression of antioxidant enzymes [69,70]. In addition, CS treatment increased the activity of antioxidant enzymes, including catalase and SOD, in animal models of spinal cord injury [16], bleomycin-induced lung injury [62] and chronic stress-related brain damage [63]. Taken together, our findings suggest that CS regulates antioxidant enzymes to inhibit UUO-induced oxidative stress.

ER stress occurs when the capacity of ER to fold proteins is exceeded and plays an important role in the pathogenesis of many diseases, including CKD [40,71]. Previous studies have shown induction of ER stress and the subsequent unfolded protein response (UPR) in the UUO model [72,73]. In this study, CS treatment inhibited ER stress in kidneys of UUO mice, as evidenced by reduced mRNA or protein expression of UPR signaling molecules (GRP78, IRE1α, PERK, ATF4, ATF6, CHOP, XBP1s and p-eIF2α). Consistently, CS inhibited ER stress in intestinal epithelial cells and maintained intestinal barrier function in an animal model of colitis [15]. CA also suppressed ER stress in mucosal tissues of patients with ulcerative colitis [15]. Moreover, ER stress can be induced by accumulation of ROS [38,39]. It has been known that the crosstalk between oxidative stress and ER stress can further induce or exacerbate oxidative stress [39]. NOX4 plays a role in mediating the interplay between oxidative stress and ER stress [74]. NOX4-mediated ROS generation can activate UPR pathways, leading to cell death and inflammation [75,76]. NOX4 inhibition has been shown to suppress cell death and inflammatory responses via attenuating UPR pathways [77,78]. Therefore, the protective effect of CS against UUO-induced renal injury and fibrosis may be at least partially due to inhibition of NOX4-mediated ER stress.

Accumulating evidence has demonstrated that tubular injury causes interstitial fibrosis, capillary rarefaction, and glomerulosclerosis, suggesting that damaged tubular epithelium plays a direct and important role in the pathophysiology of CKD [42,43]. Inhibition of tubular cell apoptosis ameliorated interstitial fibrosis in UUO mice [79,80]. In addition to apoptosis, necroptosis has recently been shown to play an important role in organ fibrosis and has been proposed as a potential target for anti-fibrotic therapies [45]. Necroptosis is a programmed necrotic cell death and is regulated by RIPK1-RIPK3-MLKL signaling cascade [45]. A previous study showed that necroptosis plays a more significant role in mediating tubular cell injury than apoptosis in the subtotal nephrectomy model of CKD [81]. Moreover, recent studies have shown that inhibition of necroptosis attenuated UUO-induced renal interstitial fibrosis and inflammation [82,83]. In this study, CS treatment remarkably inhibited UUO-induced apoptosis and necroptosis, as evidenced by a decrease in the number of TUNEL-positive cells and the expression of key factors related to apoptosis (cleaved caspase-3, cleaved PARP-1, p53 and Bax) and necroptosis (RIPK1, RIPK3 and MLKL). Because both types of cell death can be induced by ER stress [84,85], the interplay between oxidative stress and ER stress can cause or exacerbate tubular cell apoptosis and necroptosis in UUO mice. Therefore, suppression of oxidative stress induced by CS may inhibit apoptosis and necroptosis through inhibiting UPR pathways.

In response to renal injury, inflammation initially acts as a protective response, but prolonged inflammation can promote the fibrotic process [46]. The inflammatory response in UUO mice is characterized by pro-inflammatory cytokine production and immune cell infiltration [21,22]. During necroptosis, intracellular components are released from dying cells and trigger an innate immune response [45]. In this study, CS treatment reduced serum and renal levels of TNFα, IL-6 and IL-1β with inhibition of IκBα/NFκB cascade in UUO mice. Consistently, Cs has been reported to attenuate LPS-induced cytokine production in cardiomyoblasts by inhibiting the NFκB pathway [86]. Schwager et al. also showed that CS inhibits cytokine production and NFκB activation in murine macrophages and human chondrocytes [87]. In addition to its in vitro effects, CS also reduced serum or tissue levels of cytokines with inhibition of the NFκB pathway in animal models of inflammatory diseases such as spinal cord injury [16], atopic dermatitis [19] and rheumatoid arthritis [88]. In this study, we also found that CS suppressed infiltration of neutrophils and macrophages in kidneys of UUO mice, as indicated by reduced MPO activity and decreased numbers of Ly6B.2-positive cells and F4/80-positive cells. Consistently, CS reduced expression of the chemokines CXCL5 and MCP-1 in kidneys of UUO mice. The adhesion molecules ICAM-1 and VCAM-1 were also downregulated by CS. Previous studies have shown that both immune cells play an important role in UUO-induced renal injury and fibrosis [89,90]. Immune cells, including neutrophils and macrophages, produce many pro-fibrogenic cytokines that induce the accumulation and activation of myofibroblasts, resulting in excessive production of ECM [43].

## 5. Conclusions

In conclusion, our data show that CS treatment ameliorates renal injury and fibrosis in the UUO model. These effects of CS were accompanied by suppression of oxidative stress, tubular cell death and inflammation. The inhibitory effect of CS on oxidative stress was mediated by the regulation of pro-oxidant and antioxidant enzymes. These results suggest that CS might be a potential therapeutic agent for renal fibrosis.

## Figures and Tables

**Table 1:** List of primers.

Gene	Primer Sequence (5′→3′)	Accession No.
*NGAL*	F: GGCCAGTTCACTCTGGGAAA R: TGGCGAACTGGTTGTAGTCC	NM_008491
*KIM-1*	F: ACATATCGTGGAATCACAACGAC R: ACTGCTCTTCTGATAGGTGACA	NM_134248
*Fibronectin*	F: CGAGGTGACAGAGACCACAA R: CTGGAGTCAAGCCAGACACA	NM_010233
*TGF-β1*	F: GCCCTGGATACCAACTATTGCTT R: AGTTGGCATGGTAGCCCTTG	NM_011577
*CTGF*	F: CAAAGCAGCTGCAAATACCA R: AGTGGAGCGCCTGTTCTAAG	NM_010217
*Vimentin*	F: GATCGATGTGGACGTTTCCAA R: GTTGGCAGCCTCAGAGAGGT	NM_011701
*α-SMA*	F: ACTACTGCCGAGCGTGAGAT R: AAGGTAGACAGCGAAGCCAG	NM_007392
*N-cadherin*	F: AGCGCAGTCTTACCGAAGG R: TCGCTGCTTTCATACTGAACTTT	NM_007664
*E-cadherin*	F: CAGGTCTCCTCATGGCTTTGC R: GGTAGCCAGTGAGCTGAACAC	NM_009864
*NOX4*	F: CCCAAGTTCCAAGCTCATTTCC R: TGGTGACAGGTTTGTTGCTCCT	NM_015760
*5-LOX*	F: ATTGTTCCCATTGCCATCCAGCTCA R: TCGTTCTCATAGTAGATGCTCACCA	NM_009662
*XO*	F: CAGGGTCTTGGTCTTTTCAC R: CGTTGGTTTCAGCGTCAGGA	NM_011723
*COX-2*	F: AACCGCATTGCCTCTGAAT R: CATGTTCCAGGAGGATGGAG	NM_011198
*iNOS*	F: CGAAACGCTTCACTTCCAA R: TGAGCCTATATTGCTGTGGCT	NM_010927
*Catalase*	F: CAAGTACAACGCTGAGAAGCCTAAG R: CCCTTCGCAGCCATGTG	NM_009804
*Mn* *SOD*	F: AACTCAGGTCGCTCTTCAGC R: CTCCAGCAACTCTCCTTTGG	NM_0136671
*GP* *X1*	F: GCAATCAGTTCGGACACCAG R: CACCATTCACTTCGCACTTCTC	NM_008160
*PRDX5*	F: CGGAAAGAAGCAGGTTGGGA R: CATCTGGCTCCACGTTCAGT	NM_012021
*GRP78*	F: TGGTATTCTCCGAGTGACAGC R: AGTCTTCAATGTCCGCATCC	NM_001163434
*IRE1α*	F: GCATCACCAAGTGGAAGTATC R: ACCATTGAGGGAGAGGCATAG	NM_023913
*PERK*	F: AAAAAGCAGTGGGATTTGGA R: CTGGAATATACCGAAGTTCAAAG	NM_
*ATF4*	F: GAGCTTCCTGAACAGCGAAGTG R: TGGCCACCTCCAGATAGTCATC	NM_009716
*ATF6*	F: CCCAAGCTCTCCGCATAGTC R: TAAAATGCCCCATAACTGACCAA	NM_001081304
*CHOP*	F: GTCCCTAGCTTGGCTGACAGA R: TGGAGAGCGAGGGCTTTG	NM_007837
*TNF-α*	F: CACAGAAAGCATGATCCGCGACGT R: CGGCAGAGAGGAGGTTGACTTTCT	NM_013693
*IL-6*	F: TAGTCCTTCCTACCCCAATTTCC R: TTGGTCCTTAGCCACTCCTTC	NM_031168
*IL-1* *β*	F: CGCAGCAGCACATCAACAAGAGC R: TGTCCTCATCCTGGAAGGTCCACG	NM_008361
*CXCL5*	F: TCATGAGAAGGCAATGCT R: ACATTATGCCATACTACGAAGA	NM_009141
*MCP-1*	F: TAAAAACCTGGATCGGAACCAA R: GCATTAGCTTCAGATTTACGGGT	NM_011333
*ICAM-1*	F: AACTGTGGCACCGTGCAGTC R: AGGGTGAGGTCCTTGCCTACTTG	NM_010493
*VCAM-1*	F: CCCAGGTGGAGGTCTACTCA R: CAGGATTTTGGGAGCTGGTA	NM_011693
*GAPDH*	F: ACTCCACTCACGGCAAATTC R: TCTCCATGGTGGTGAAGACA	NM_001289726

## Data Availability

The data supporting the findings of this study are available within the article.
